# Advances in Cardiopulmonary Life-Support Change the Meaning of What It Means to be Resuscitated

**DOI:** 10.1089/pmr.2020.0002

**Published:** 2020-06-01

**Authors:** Leslie C. Avant, Carolyn E. Kezar, Keith M. Swetz

**Affiliations:** ^1^ Department of Medicine, University of Alabama at Birmingham, Birmingham, Alabama, USA.; ^2^ Department of Medicine, Division of Gerontology, Geriatrics, and Palliative Medicine, University of Alabama at Birmingham, Birmingham, Alabama, USA.; ^3^ Birmingham Veterans Affairs Medical Center, Birmingham, Alabama, USA.

**Keywords:** end of life, palliative care, patient–physician relationship, withholding, withdrawing

## Abstract

As options for advanced cardiopulmonary support proliferate, the use of mechanical circulatory support, such as left ventricular assist device as destination therapy (LVAD-DT), is becoming increasingly commonplace. In the current case, a patient was hospitalized for complications related to his LVAD-DT requests “full code” status, despite a clinician's warning that performing chest compressions may damage the LVAD device or vascular structures leading to poor outcome. This discussion explores the ethical and legal considerations regarding a patient request for cardiopulmonary resuscitation when limited options for survival or further treatment are available.

## Case

Mr. T is a 35-year-old man with a history of nonischemic cardiomyopathy. About three years prior, he received a left ventricular assist device (LVAD) as a bridge-to-transplantation. However, due to obesity and limited social support, he is no longer deemed a suitable candidate for cardiac transplantation. Mr. T experiences multiple complications including driveline infections, arrhythmias, and, now, he has developed a pump thrombosis. Having previously received a pump exchange and now no longer being considered a transplantation candidate, Mr. T is deemed too high risk for surgical intervention. While hospitalized with hemolysis and worsening cardiac output consistent with pump thrombosis despite receiving multiple attempts at antithrombotic therapy, Mr. T requests “full code” resuscitation status. He states that he refuses “to give up,” and wishes to focus on hope that current antithrombotic therapies “will work.” The clinician (a certified nurse practitioner with expertise in cardiac care) explains that chest compressions may dislodge the LVAD, causing bleeding into the chest cavity. She then asks whether the patient “really wants chest compressions?” Emphatically, he requests “full code” status, despite the clinician's concerns about any efficacy of cardiac resuscitation beyond transient support. The patient is also requesting mechanical ventilator support if needed. Palliative care team was consulted to assist with goals of care clarification.

## Discussion

With the tremendous advances in cardiopulmonary care, utilization of an LVAD as destination therapy (LVAD-DT) has increased due to its provision of improved survival and quality of life for many patients. DT refers specifically to patients who receive LVAD support and who do not desire or meet criteria for heart transplantation. As of 2019, >2600 LVADs were implanted (or projected to be implanted) annually in the United States.^1^ Although ∼50% of LVADs implanted from 2015 to 2018 were done as DT, this number increased to ∼70% of total implantations in 2019.^1^ Furthermore, it is estimated that as many as 150,000 to 250,000 patients may qualify for implantation in the future.^2^ Compared to patients with advanced heart failure without LVAD, most patients with LVAD-DT die in the hospital—many in the intensive care setting.^3,4^ Patients who survive may experience complications of infection, stroke, bleeding, or persistent right heart failure, which negatively affect quality of life and independence.^5^ However, relative rates of complications vary somewhat by device type and its functional characteristics.

Ethical challenges can arise regarding end-of-life care that clinicians report feeling ill-equipped to handle.^6^ Even if clinicians are comfortable addressing such situations, differing opinions among stakeholders may lead to uncertainty.^7^ In this case, the patient's request for ongoing treatment (respect for autonomy) and what is deemed medically appropriate (beneficence, nonmaleficence, and justice) did not align. In such cases, how is the clinician obligated to respond?

### Weighting benefits versus burdens

Beauchamp and Childress remind us of the *prima facie* duties clinicians have, in that principles of biomedical ethics are nonhierarchical and require weighing and balancing through the process of specification.^8^ No single principle trumps all. Respect for autonomy requires physicians to attend to patients' preference regarding their medical care, ensuring patients have an adequate understanding of specific medical inventions, while enabling them to make informed decisions.^9^ As with LVAD implantation, patients and physicians should engage in collaborative and shared decision making, and patient preferences should be honored when possible.^10,11^ Nonetheless, it has been argued that strict respect for autonomy weakens consideration of other principles.^12^

Consent to cardiopulmonary resuscitation (CPR) is presumed and considered the standard of care in a hospital or in the community. To avoid undergoing CPR, patients must explicitly declare their decision to opt out of the intervention when cardiac or pulmonary function ceases in the future. When a patient with intact decision-making capacity (or a duly executed advance directive) invokes the right to refuse CPR, it *must* be honored. Conversely, considering the default right to remain “full code” despite concerns that CPR (or some component thereof) is not medically appropriate can be morally distressing for clinicians and merits further discussion. Through the process of specification, one could argue that although the patient does not have an ethical right to cardiac resuscitation against the objection of providers, his interests and autonomous choices are relevant to code status decisions. However, the do-not-resuscitate (DNR) order is currently so strongly embedded within the patient autonomy paradigm that one author has considered it a “patient order” that is given to the medical team,^13^ leaving little opportunity for other ethical principles to be actualized.^14,15^

In consideration of the principles of beneficence and nonmaleficence, it is important to weigh the effectiveness and harms of attempted cardiac resuscitation. Studies have noted that efficacy of CPR in treating in-hospital cardiac arrest is ∼17%,^16,17^ with variability by comorbid conditions. For example, outcomes of CPR in the setting of malignancy,^18^ dementia,^19^ concurrent hemodialysis,^20^ or sepsis are far worse than outcomes associated with witnessed arrest in an ICU setting or cardiac arrest due to acute coronary syndrome.^17,19^ It is important to consider that, as in the current case, patients with an LVAD have advanced Stage D heart failure and often have comorbidities that have precluded heart transplantation.^21^ No large scale studies have addressed mortality outcomes after CPR in patients with an LVAD. However, one small single-center study showed that 31% of 16 patients who had a cardiopulmonary arrest with an LVAD survived to discharge. However, no further data were provided as to whether these patients had long-term disability or sequellae because of their arrest or use of CPR.^22^

Moreover, performing cardiac resuscitation in LVAD patients presents multiple concerns. Standard Advanced Cardiovascular Life Support (ACLS) guidelines do not readily apply to LVAD patients, and an LVAD in and of itself is a form of constitutive resuscitation beyond what is included in traditional CPR decisions. As noted in the case, potential harms of chest compressions include risk of damage to the LVAD itself, device connections, or anastomoses, which may result in massive hemorrhage. Furthermore, LVAD patients frequently do not have a palpable arterial pulse or easily measurable blood pressure if pulse pressure drops substantially.^23^ As a result, health care providers may be uncertain about the safety and role of ACLS in patients with an LVAD, and there may be concerns about when to begin resuscitation attempts to avoid potential hazards of inappropriate attempts. The American Heart Association has published consensus guidelines that contain an algorithm for approaching ALCS and resuscitation in the setting of an LVAD, and that acknowledge and attempt to address these potential harms.^24^ As a general rule, the guidelines state the harm of withholding chest compressions is noted to outweigh the potential for dislodging the device if circulatory failure is not attributable to device failure.^24^ However, in the present case, the pump has thrombosed and is malfunctioning as evidenced by hemolysis, increased power consumption, and decreasing cardiac output. Reversible causes of cardiac arrest are very unlikely given the patient's clinical situation.

### To what end?

In our patient's situation, there remains one critical question: even if the patient has a cardiac arrest and receives cardiac resuscitation with return of spontaneous circulation (ROSC), what is the endpoint? The long-term prognosis of patients with an LVAD-DT continues to be relatively limited, with estimates of three-year survival after implantation at 59%.^1^ In the present case, the patient does not qualify for heart transplantation, nor is he eligible for extracorporeal membrane oxygenation or a new LVAD implantation. In essence, these are the only treatments that could be life prolonging and adequately treat his current condition. However, he is deemed too high risk to survive any of these options. Assuming that ROSC occurs, no further means of supporting circulation is available given the pump thrombosis and a machine that essentially is no longer functional. To this end, the patient is dying from an irreversible condition. Resuscitation, in these cases becomes a “pseudo-option”^13^ because it is not possible to return patients to a satisfactory level of health, or in this case—prolong survival or return to state of wellness. We believe that acceding to the patient's request without a reasonable chance of achieving and maintaining ROSC would not only be unethical based upon the principle of nonmaleficence, but it would also be allowing the patient to operate under the illusion that such intervention may be successful.

Reiterating from earlier, in most U.S. health care environments, if the patient does not request a DNR order, an implied “order” to perform CPR is presumed to exist, regardless of whether such procedure is medically indicated.^13^ However, we contend that for patients with an LVAD, such an implied “full code” order may or may not be appropriate and warrants further discussion in the literature. In the current case, an existing pump thrombosis makes it unreasonable to expect external chest compressions to benefit the patient. Therefore, the interpretation that a patient (or patient's surrogate) must always consent to a DNR order is misguided—not only because it eliminates the professional purview of the physician, but also because it violates the principle of nonmaleficence by overly emphasizing patient autonomy.

Legally, it has been determined that a patient has the right to decline medical treatment. However, there is no consensus on whether a patient has a legal right to demand a life-sustaining intervention when the physician believes it is unlikely to be of benefit.^25^ In the United States, courts have been inconsistent when posed with the issue of physician–patient disagreement, and state statutes vary widely regarding a physician's right to refuse a patient's request for treatment.^13^ Although most states generally grant the physician the right to refuse “medically inappropriate” treatment, statutes generally fall short of defining this term.^26^ Further there is often no explicit criteria provided for how a physician may refuse. Exceptions to this generalization are provided by Texas, Virginia, and New York. The Texas Advance Directives Act permits an attending physician to refuse to honor a patient's (or health care proxy's) request for life-sustaining treatment based on the physician's clinical judgment, although this decision must be reviewed by an institutional ethics committee or medical review board.^27^ Similarly, the Virginia Code provides a provision negating the physician responsibility of providing “medically ineffective” treatment, and allows the determination of medical ineffectiveness to be made by two physicians (or one if the patient is being treated in an emergency department and a second physician is not available).^28^

New York provides a stark contrast to the statutes of Texas and Virginia, and denies a physician the right to refuse a patient's request for treatment. Although New York's “DNR law” was intended initially to provide clinicians with the authority to implement a DNR order, it was later updated stating that a physician is required to obtain the consent of the patient's health care surrogate before issuing a DNR order, even in instances when the administration of CPR would be “medically futile.”^12^ Thus in the present case, where an existing LVAD device with pump thrombosis makes the possibility of cardiac resuscitation an ethical “pseudo-option,” in most states it remains unclear whether the clinician would have legal basis for refusal. However, New York would require the patient to remain “full code” despite the physician assertion that this is not medically advisable.

### Back to the case

Returning to the *prima facie* duties of clinicians, when the potential harms of an intervention far outweigh any benefits or no benefits exist (as in the current case), we contend that the clinician has a moral responsibility to object to the provision of such a treatment, regardless of the demand of the patient. In this instance, with the help of our palliative care team, the heart failure team and the patient engaged in a frank discussion regarding the fact that cardiac resuscitation would not change the patient's outcome. Although the patient acknowledged that he was dying, he did not wish to have discussions regarding his terminal state, and our attempts at educating him around the lack of benefit to cardiac resuscitation were ultimately unsuccessful. It was determined that the patient would remain “full code” but, in the instance of LVAD stoppage or sustained ventricular arrhythmias, chest compressions were not medically advisable as there were no sustainable interventions to restore circulation in this patient. Any attempt to perform external chest compressions would risk damage to the device, its anastomoses, or could possibly result in hemorrhage or other mechanical complications. Moreover, CPR would not allow for temporizing of his condition or for other options to life-prolonging treatment to become available.

Ultimately, despite attempts at multimodal anticoagulation, the patient's condition worsened with progression to disseminated intravascular coagulation and LVAD stoppage with cardiogenic shock. The patient quickly became apneic (within five minutes after LVAD cessation). The patient quickly progressed to asystole with family at bedside. They grieved appropriately and understood that this represented the patient's end of life and supported measures for comfort. No chest compressions or vasoactive medications were administered, and the patient died peacefully seven minutes after LVAD cessation.

## Conclusion

Current cardiopulmonary technologies have created situations wherein recovery or survival is not conceivable in some instances. How best to proceed when this occurs can be particularly challenging for clinicians who must weigh what options best respect patient autonomy and what options legitimately respect beneficence and nonmalaficience. As technology evolves, decisions about CPR and resuscitation may have to be made in circumstances that do not fit within the typical paradigm for our understanding of CPR. Although CPR was developed to attempt to restore spontaneous circulation in situations wherein there was a “next step” toward maintaining that circulation, the default opt-in for CPR can create clinical challenges in groups wherein that intended goal of maintaining circulation is just not feasible. In these situations, we believe the medical community must support clinicians and colleagues who struggle in caring for patients with complex care needs that fall outside of the traditional realm of what one considers to be resuscitation.

## Abbreviations Used

ACLS: advanced cardiovascular life support
CPR: cardiopulmonary resuscitation
DNR: do-not-resuscitate
LVAD-DT: left ventricular assist device as destination therapy
ROSC: return of spontaneous circulation
